# Tumor-derived TGF-β and prostaglandin E2 attenuate anti-tumor immune responses in head and neck squamous cell carcinoma treated with EGFR inhibitor

**DOI:** 10.1186/s12967-014-0265-3

**Published:** 2014-09-21

**Authors:** Takumi Kumai, Kensuke Oikawa, Naoko Aoki, Shoji Kimura, Yasuaki Harabuchi, Esteban Celis, Hiroya Kobayashi

**Affiliations:** Department of Pathology, Asahikawa Medical University, Asahikawa, Japan; Department of Otolaryngology, Head and Neck Surgery, Asahikawa Medical University, Asahikawa, Japan; Cancer Immunology, Inflammation and Tolerance Program, Georgia Regents University Cancer Center, Augusta, GA USA

**Keywords:** HNSCC, EGFR, TGF-β, COX-2

## Abstract

**Background:**

EGFR-targeted therapy is an attractive option for head and neck squamous cell carcinoma patients. We have recently reported the use of EGFR inhibitors as an adjunct treatment to enhance HLA-DR expression in tumor cells to improve cancer immunotherapy. Nevertheless, we observed that EGFR inhibitors resulted in decreased anti-tumor responses, regardless of upregulation of HLA-DR expression on the tumor cell. In this study, we specifically investigated the mechanisms by which EGFR inhibition modulated anti-tumor responses.

**Methods:**

An EGFR inhibitor erlotinib was used to assess the modulation of anti-tumor responses by tumor antigen-specific helper T cells. We then examined whether administration of the EGFR inhibitor altered tumor cytokine profiles and expression of immune-related molecules on tumor cells.

**Results:**

Despite the augmented HLA-DR expression on a gingival cancer cell line by EGFR inhibition, anti-tumor responses of EGFR reactive helper T cell clones against tumor cells were decreased. EGFR inhibition did not change the expression of CD80, CD86, or PD-L1 on the tumor cells. Conversely, production of transforming growth factor beta (TGF-β) and prostaglandin E2 was increased by EGFR inhibition, indicating that these immunosuppressive molecules were involved in diminishing tumor recognition by T cells. Significantly, attenuation of HTL responses against tumors after EGFR inhibition was reversed by the addition of anti-TGF-β antibody or COX2 inhibitors.

**Conclusions:**

Targeting TGF-β and prostaglandin E2 may allow for improved outcomes for cancer patients treated with combination immunotherapy and EGFR inhibitors.

## Background

Immunotherapy is a promising strategy for increasing survival in cancer patients, and has been an active area research for decades. Amongst various types of immunotherapy, the induction of anti-tumor CD8 cytotoxic T lymphocyte (CTL) responses via vaccination with peptide epitopes has been widely applied in the clinical setting [1]. Unfortunately, CTL vaccines have not yet yielded clear favorable clinical results for treating cancer, possibly due to a combination of suboptimal immune responses and to tumor-derived immune suppressive activities.

Many strategies have been applied to enhance antigen-specific anti-tumor immunity, including the activation of natural killer (NK) cells, conversion of macrophage phenotype, and immune-modulating adjuvants [2-4]. Among these, the blockade of immunological checkpoints such as CTLA-4/B7 and PD-1/PD-L1 is quite advanced and has yielded promising clinical results [5]. It is predicted that the use of non-specific anti-cancer immunity targeted therapy may be a valuable complement to tumor antigen-specific immunity against cancer.

CD4^+^ helper T lymphocytes (HTLs) play a critical role in anti-cancer immunity by promoting the induction and survival of CD8^+^ CTLs. In addition, in some instances HTLs can also exhibit direct anti-tumor cytotoxic activity. In view of this, our laboratories have focused on the identification of peptide epitopes capable of inducing cytotoxic HTL responses against tumor cells that express surface MHC class II molecules [6]. Recently, long-peptide vaccines have been used with the purpose of inducing both CTL and HTL anti-tumor responses, with promising clinical results [7].

The disruption of the antigen-processing machinery is one of the mechanisms utilized by tumor cells to evade T cell recognition. To overcome this problem, we and other groups have recently proposed that the increase of MHC class II protein expression on tumor cells obtained with EGFR inhibitors could be implemented to enhance HTL anti-tumor responses [8,9]. Although EGFR inhibitors have been widely used to treat many types of cancer, the usefulness of these therapies is limited due to the appearance of drug resistance [10,11]. Immune regulatory cytokine TGF-β has been reported to mediate the resistance to EGFR inhibition, however, direct activity of EGFR mediated TGF-β production from tumor toward antitumor immune cells has remained largely unknown [12].

In this study, we discovered that EGFR inhibition although increased MHC-II expression, paradoxically it attenuated HTL responses against some head and neck squamous cell carcinoma (HNSCC) cells. We observed that secretion of TGF-β and PGE2 by the HNSCC cells was increased following EGFR inhibition, despite a lack of evident changes in immune costimulatory molecules or EGFR expression in tumor cells. Inhibition of TGF-β or COX-2 salvaged HTL responses against EGFR inhibitor-treated HNSCC cells, suggesting that these pathways played a crucial role in immunosuppression. Taken together, our results demonstrate that in some cases, EGFR inhibitors may skew the immune response towards T cell suppression, and that concomitant blockade of EGFR and TGF-β/COX-2 may be promising combinatorial therapeutic approaches for patients with EGFR-expressing tumors.

## Materials and methods

### Cell lines

HNSCC cell lines HSC-3, HSC-4 (tongue SCC, DR1/4) and Sa-3 (gingival SCC, DR9/10) were provided by the RIKEN Bio-Resource Center (Tsukuba, Japan). CA9-22 (gingival SCC) and HPC-92Y (hypopharyngeal SCC) were kindly provided by Dr. Yasuharu Nishimura (Dep. of Immunogenetics, Kumamoto University, Kumamoto, Japan) and Dr. Syunsuke Yanoma (Yokohama Tsurugamine Hospital, Yokohama, Japan), respectively. SAS (tongue SCC), Calu-1 (non-small cell lung carcinoma) and 5637 (bladder cancer) were purchased from American Type Culture Collection (Manassas, VA). All cell lines were maintained in RPMI 1640 (nacalai tesque, Kyoto, Japan) supplemented with 10% fetal bovine serum.

### Western blotting

Cells (1 × 10^6^) were washed in phosphate-buffered saline (PBS) and lysed in NuPAGE sample buffer (Invitrogen, CA). The lysates were subjected to electrophoresis (NuPAGE bis-Tris SDS-PAGE gel (Invitrogen, CA)) and transferred to Immobilon-P membrane (Millipore, Bedford, MA). The membrane was soaked in blocking buffer (PBS containing 5% non-fat dry milk and 0.01% Tween 20, 1 h) at room temperature. Blots were then incubated with polyclonal rabbit anti-human EGFR (sc-03; Santa Cruz Biotechnology, Inc., Santa Cruz, CA), polyclonal rabbit anti-human phospho-EGFR (Tyr1068; Cell Signaling Technology, Denver, MA), polyclonal rabbit anti-human heat shock protein 70 (HSP70) (Enzo Life Sciences, Inc., Farmingdale, NY), or monoclonal rat anti-human heat shock protein 90 (HSP90) (Enzo Life Sciences) diluted 1:500 in blocking buffer, or anti β-actin mAb (Santa Cruz Biotechnology) diluted 1:1,000 in blocking buffer, for 18 h at 4°C. The membrane was incubated with HRP-labeled sheep anti rabbit or anti mouse IgG after washing, and made visible by an enhanced chemiluminescence (ECL) system (Amersham, Buckinghamshire, UK).

### Synthetic peptides

The synthetic peptide used throughout this study was EGFR_875–889_ (KVPIKWMALESILHR) [8]. The peptide epitope was synthesized by solid-phase organic chemistry and purified by high performance liquid chromatography. The purity (80%<) and identification of peptides were assessed by high performance liquid chromatography and mass spectrometry, respectively.

### Measurement of antigen-specific responses with antigen reactive CD4^+^ T-cell clones

Antigen-specific CD4^+^ T cells were induced by peptide stimulation of fresh peripheral blood mononuclear cells (PBMCs) from healthy volunteers [13]. EGFR_875–889_-reactive CD4^+^ T cell clones T8 (from an HLA-DR 9/12 individual) and M8 (from an HLA-DR 9/13 individual) were used. These clones were restricted by HLA-DR53 as recently described [8]. CD4^+^ T-cells (3 × 10^4^ cells/well) were mixed in 96 well culture plates with irradiated antigen-presenting cells (APCs) that consisted of either autologous PBMCs (1 × 10^5^ cells/well) or tumor cell lines (3 × 10^4^ cells/well). HNSCC cells were pretreated with interferon gamma (IFN-γ, 500 U/ml, 48 h) to increase HLA-DR expression prior to the assay. To examine the role of EGFR inhibitor in augmenting the MHC class II molecules expression, HNSCC cells were preincubated with reversible EGFR tyrosine kinase inhibitor (TKI) erlotinib (1 μM; Selleck Chemicals, Houston, TX), for 2 h at 37°C before addition of IFN-γ. DMSO was used as control. Tumor cells were washed twice with PBS to eliminate the residual chemicals. Expression of the HLA-DR and B7-H1 on tumors was evaluated by flow cytometry using anti HLA-DR mAb conjugated with fluorescein isothiocyanate (FITC), anti B7-H1 mAb (eBioscience, Minneapolis, MN), and anti-mouse immunoglobulin conjugated with FITC (Dako Denmark A/S, Glostrup, Denmark). Detection of surface CD80 and CD86 was carried out using unconjugated mouse anti-human CD80 IgG1 (MAB104,Immunotech, Marseille, France) and unconjugated mouse anti-human CD86 IgG2b (HA5.2B7, Immunotech, Marseille, France) followed by FITC-conjugated rabbit anti-mouse immunoglobulin antibody (1:100; Dako, Denmark A/S, Glostrup, Denmark).

Anti-TGF-β Ab (10 μg/ml; Abcam, Tokyo, Japan), celecoxib (10 μM; Sigma-Aldrich Japan, Tokyo, Japan), recombinant PGE2 (1 μM; Sigma-Aldrich Japan) or supernatant of tumor culture were added to the co-culture medium for functional studies. CD4^+^ T-cells culture supernatants were collected after 48 h to quantify antigen-induced IL-4, IL-10 or IFN-γ production using ELISA assays (BD Pharmingen, San Diego, CA). Culture supernatants from erlotinib-treated tumor cells were collected for quantification of TGF-β and PGE2 using ELISA kits (TGF-β eBioscience, San Diego, CA; PGE2: R&D Systems, Inc., Minneapolis, MN).

### Statistical analysis

All data are presented as mean ± standard deviation. In all experiments, group differences were analyzed by using the two-tailed Student’s t test and p <0.05 was considered as statistically significant.

## Results

### Downregulation of EGFR-reactive CD4^+^ T cell responses against HNSCC cells pretreated with EGFR inhibitor

**Figure 1:**
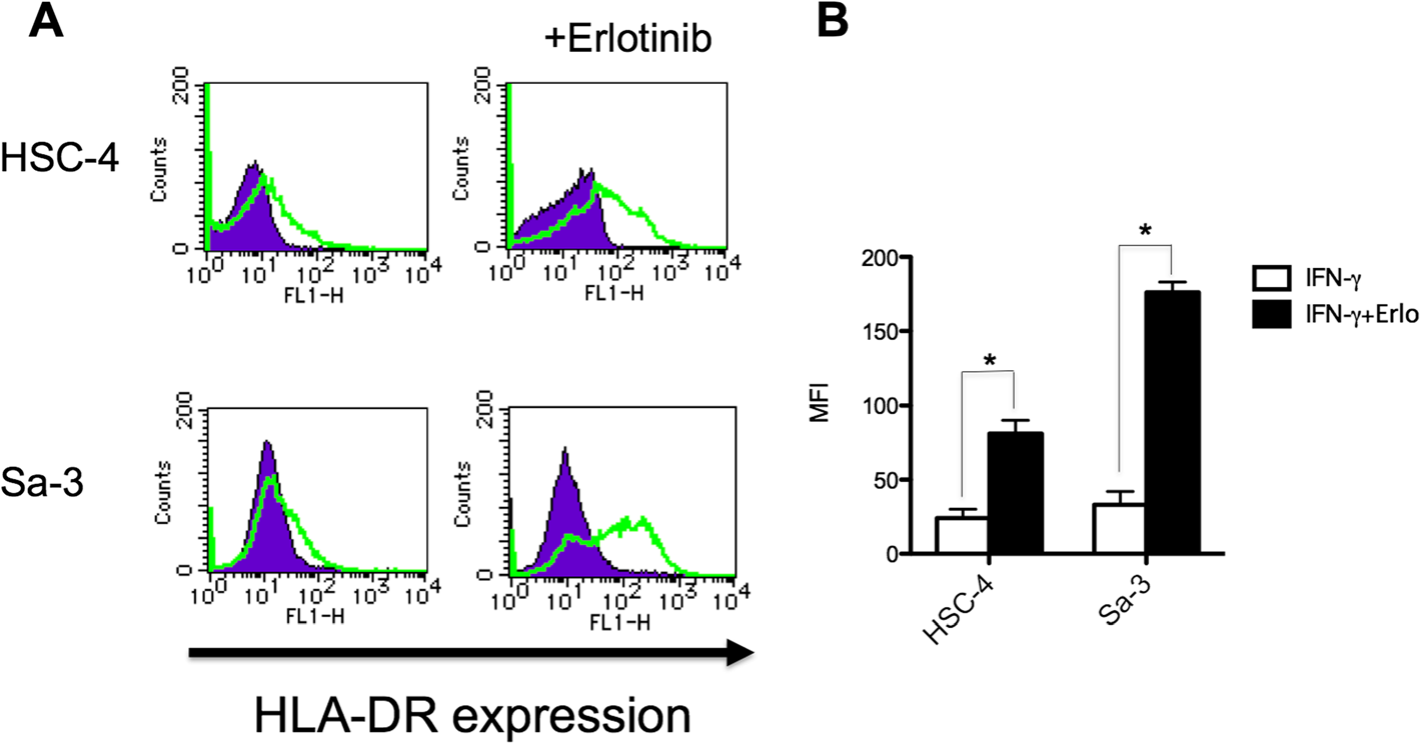
**Alteration of HLA-DR expression in HNSCCs by EGFR inhibitor. (A)** HLA-DR expression of HNSCC cell lines HSC-4 and Sa-3 was examined by flow cytometry. HNSCC cells were treated with IFN-γ (50U/ml) alone or with IFN-γ and erlotinib (1 μM) for 48 h. Purple graph: isotype control antibody, Green graph: anti HLA-DR antibody. **(B)** Mean fluorescence intensity (MFI) of HLA-DR expression was shown. Columns: means of triplicate determinations, bars: SD. Results are representative of at least two separate experiments. *p < 0.05.

**Figure 2:**
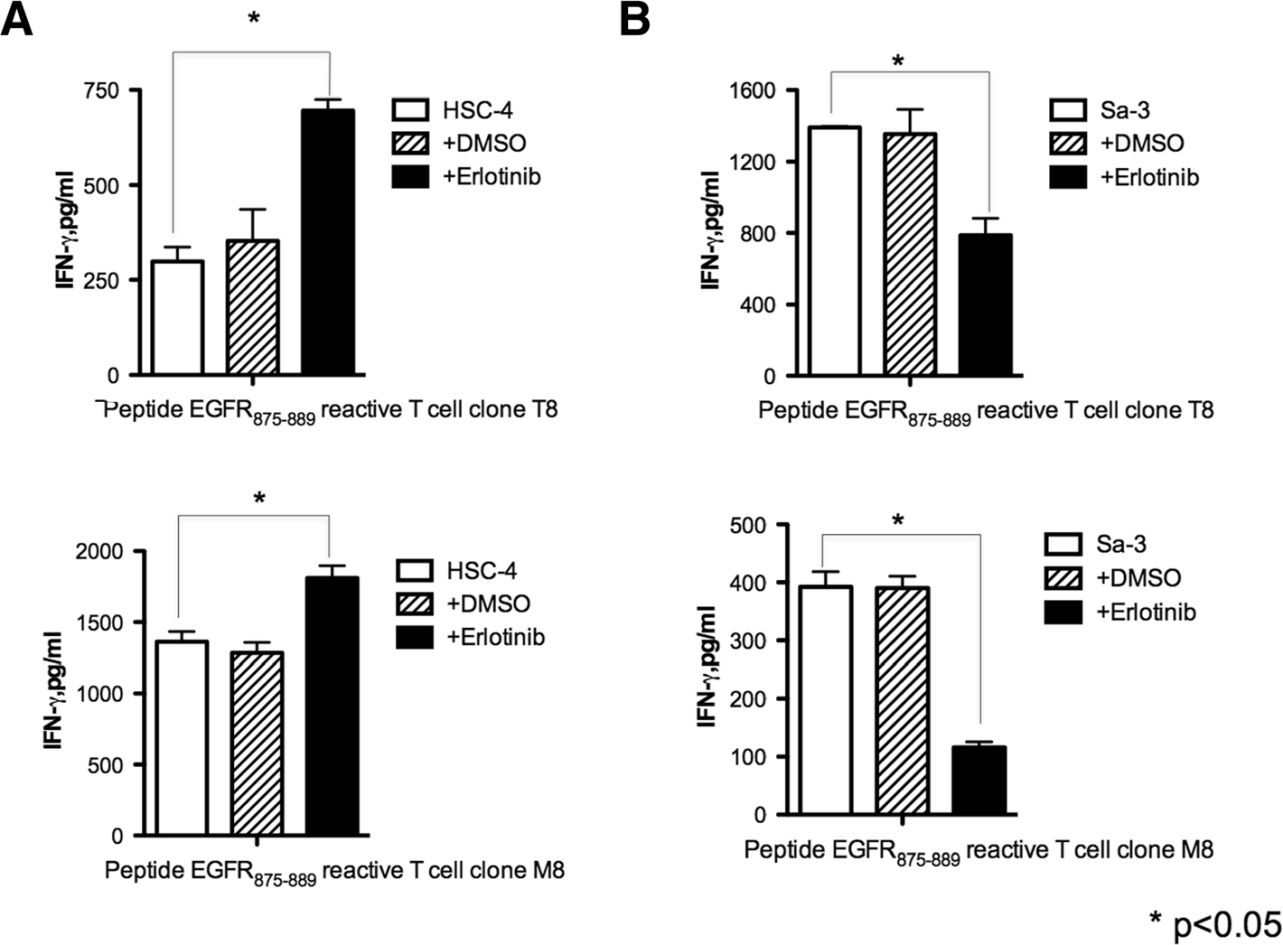
**EGFR inhibitor attenuated anti-tumor responses of EGFR**_**875–889**_ **-reactive CD4** ^**+**^ **T cell clones against Sa-3. (A) (B)** EGFR_875–889_-reactive CD4^+^ T cell clones T8 (DR53-restricted) and M8 (DR53-restricted) were tested for their capacity to recognize antigen directly on EGFR-positive, HLA-DR matched HNSCC cell line HSC-4 or Sa-3 by IFN-γ production. Tumor cells were pretreated 48 h with or without erlotinib (1 μM). DMSO was used as negative control. Columns without bars had SD of <10% of mean values. Results are representative of three separate experiments. *p < 0.05.

Recently, we reported that the newly identified CD4^+^ T cell peptide epitope EGFR_875–889_ functions as a promiscuous MHC-II HTL epitope that can elicit effective antitumor responses towards tumors expressing various HER family member proteins that have a high degree of homology in the peptide sequence [8]. In these studies we proposed that EGFR inhibitors might be effective adjuncts for cancer immunotherapy because these drugs increased MHC-II expression levels on tumor cells, which should enhance CD4^+^ T cell recognition. As shown in Figure 1A, the expression of HLA-DR in the SCC cell lines HSC-4 and Sa-3 was increased following erlotinib treatment. Moreover, the effect of erlotinib was more prominent (~2-fold higher) on the Sa-3 cells as compared to HSC-4 (Figure 1B). Because Th1 response has been reported as a key subset in antitumor T cell responses [14], we used Th1 cytokine IFN-γ as a read out for HTL reactions toward tumor or peptide stimulation. As predicted, erlotinib pretreatment enhanced HTL responses against HSC-4 (Figure 2A). In contrast, erlotinib decreased the T cell response to Sa-3 cells despite the dramatic upregulation of HLA-DR (Figure 2B). These results indicate that in addition to increasing MHC-II levels on tumor cells, EGFR inhibitors can regulate T cell recognition through other mechanisms.

### Treatment of HNSCC cells with EGFR inhibitor downregulates the expression of HSP90 but not EGFR

**Figure 3:**
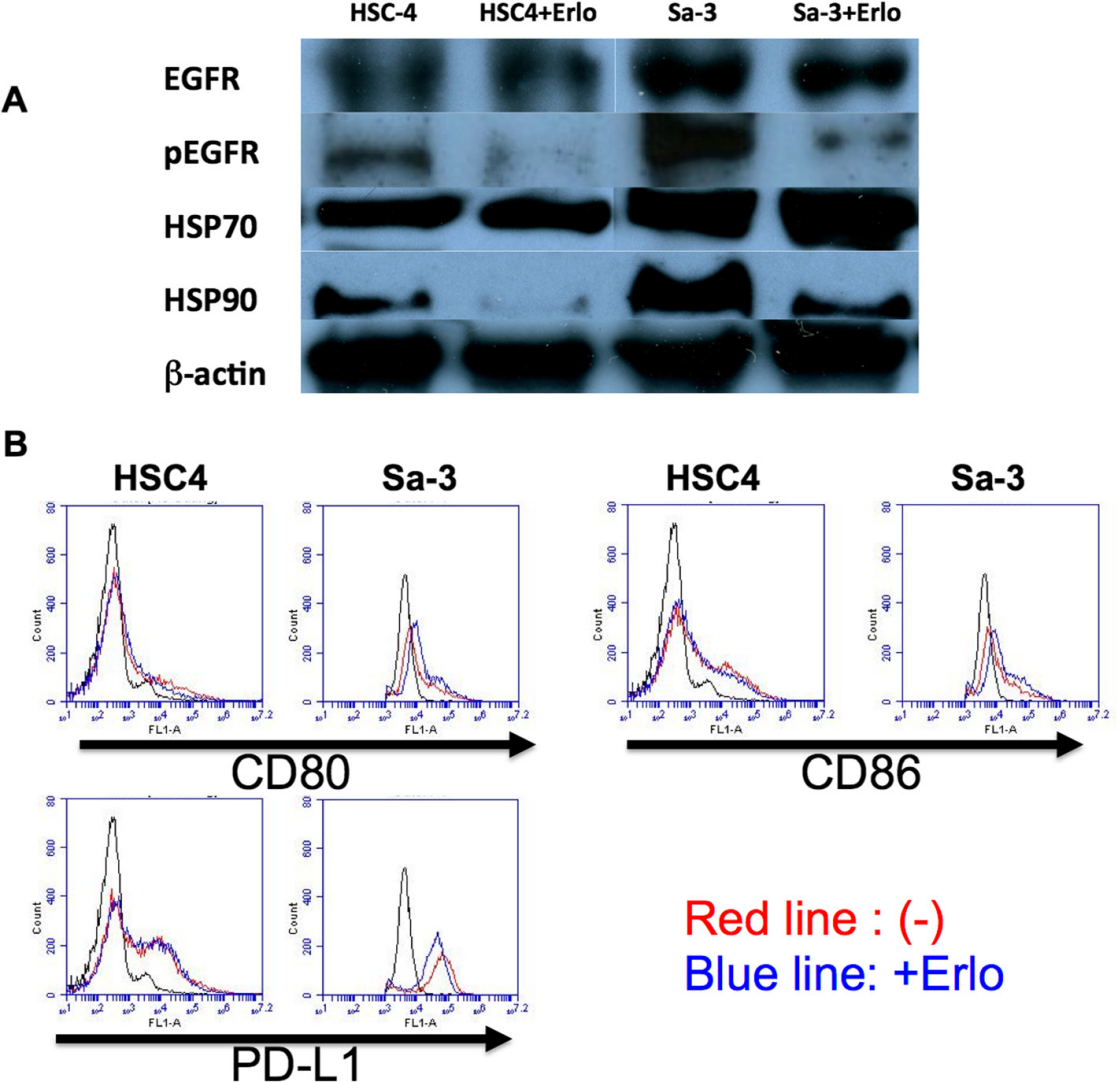
**EGFR, HSP and costimulatory molecules expression on HNSCC cells. (A)** HNSCC tumor cells were incubated with erlotinib (1 μM) for 48 h and the expression of EGFR, pEGFR, HSP70, and HSP90 was examined by western blot. **(B)** CD80, CD86, and PD-L1 expression on HNSCC cell lines HSC-4 and Sa-3 was examined by flow cytometry. HNSCC cells were treated with IFN-γ (50 U/ml) alone (red line) or IFN-γ and erlotinib (1 μM; blue line) for 48 h.

Efficient HTL responses are mediated by the interaction of T cell receptors with MHC-II molecules presenting the cognate peptide antigen on tumor cells. In turn, the levels of protein antigen made by the tumor cell sand the antigen-processing machinery will determine the production of the cognate peptide antigen. Thus, we evaluated the effect of EGFR inhibition on the levels of EGFR protein in tumor cells. As shown in Figure 3A, unlike phosphorylated EGFR, the total EGFR protein levels in HSC-4 and Sa-3 cells remained unchanged after erlotinib treatment. Meantime, we examined the expression levels of HSP70 and HSP90, which are known to be important components for antigen processing [15]. Expression of HSP70 remained unchanged by EGFR inhibition. However, HSP90 expression was decreased by erlotinib treatment, although the degree of downregulation was more prominent in HSC-4 than the Sa-3 cells (Figure 3A), which does not correlate with the observed results in HTL recognition of the tumor cells.

### Expression of costimulatory molecules on tumor cells is unaffected by EGFR inhibitor treatment

B7 family ligands are costimulatory molecules that regulate immune responses [16]. To further assess the mechanisms of the immune inhibition by erlotinib treated Sa-3 cells, we measured the surface expression of stimulatory B7 family members CD80 and CD86. After 48-h erlotinib treatment, the expression levels of both CD80 and CD86 on Sa-3 cells remained unchanged (Figure 3B). The expression level of the inhibitory molecule PD-L1 (B7-H1) on the Sa-3 tumor cells also showed no significant change after erlotinib treatment (Figure 3B). These data suggests that the effect of EGFR inhibition on the immune regulatory activity of Sa-3 was not mediated by changes in the expression of costimulatory/inhibitory B7 molecules on tumor cells.

### EGFR inhibitor promotes PGE2 and TGF-β secretion from Sa-3 tumor cells

**Figure 4:**
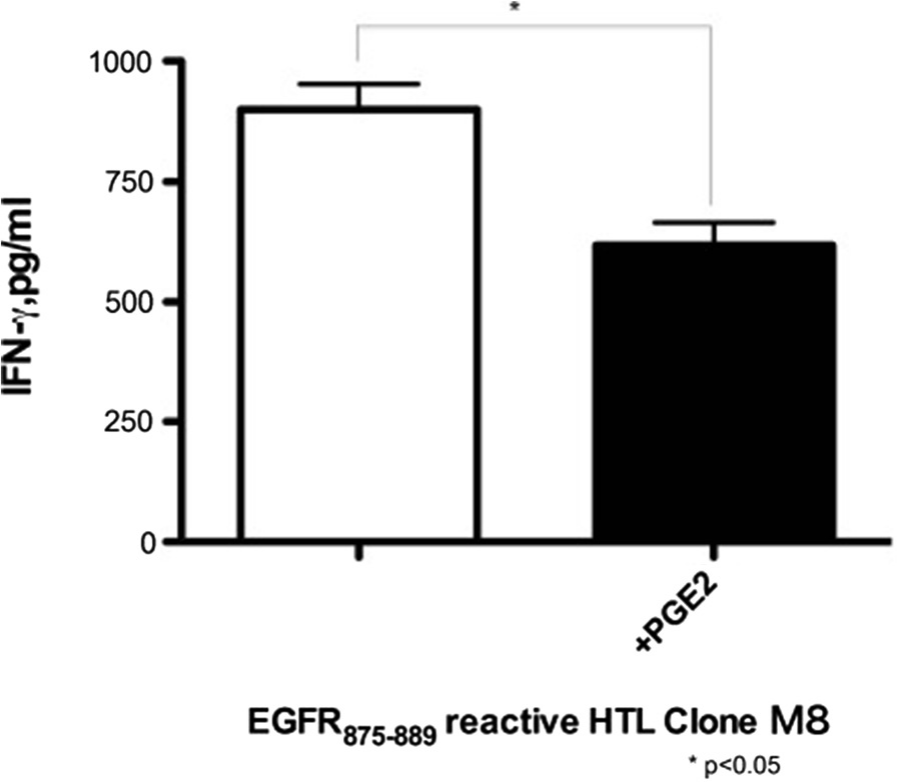
**Responses of EGFR**_**875–889**_ **-reactive CD4** ^**+**^ **T cell clones to EGFR**_**875–889**_ **peptide were attenuated by PGE2.** EGFR_875–889_-reactive CD4^+^ T cell clone M8 was tested for its capacity to recognize EGFR_875–889_ peptide by using autologous PBMCs as APCs with or without PGE2 (1 μM) by quantification of IFN-γ production. Columns without bars had SD of <10% of mean values. Results are representative of three separate experiments. *p < 0.05.

**Figure 5:**
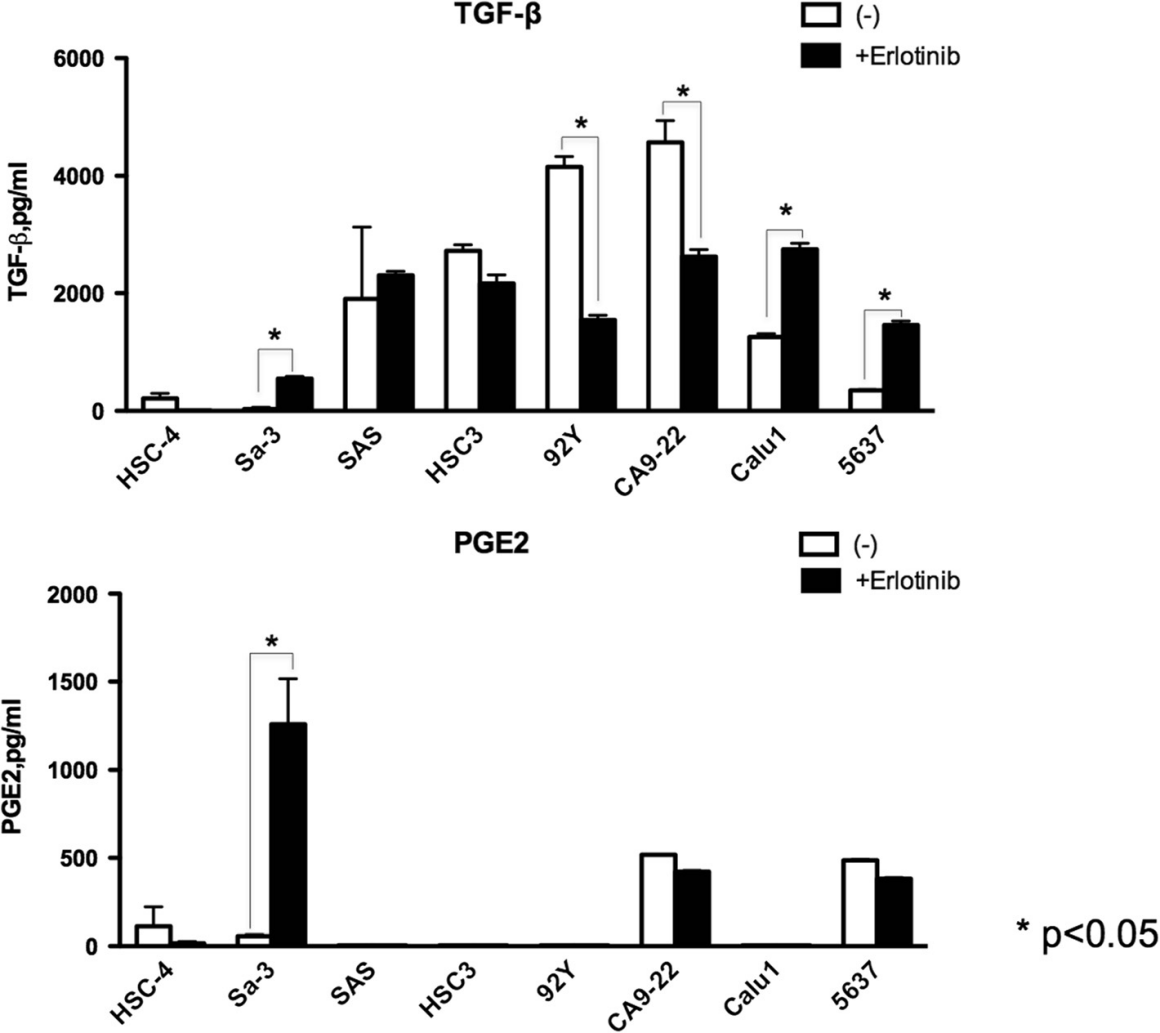
**EGFR inhibitor modulated PGE2 and TGF-β production from HNSCC cell lines.** HNSCC cell lines HSC-4, Sa-3, SAS, HSC-3, HPC-92Y and CA9-22, non-small cell lung cancer cell line Calu-1 and bladder cancer cell line 5637 were tested for their capacity to produce PGE2 and TGF-β. Tumor cells were pretreated 48 h with or without erlotinib (1 μM). Columns without bars had SD of <10% of mean values. Results are representative of three separate experiments. *p < 0.05.

Soluble factors such as cytokines and prostaglandins can regulate the immune function of T cells [17,18]. Indeed, as shown in Figure 4 the addition of PGE2 decreases the HTL response to EGFR_875–889_-loaded PBMCs as antigen-presenting cells, suggesting that the immuno-inhibitory effect on T cells could be partly due to the effects of PGE2. Thus, we evaluated whether the negative effects of erlotinib in the T cell recognition of Sa-3 were mediated by immunosuppressive factors produced by the tumor cells as the result of EGFR inhibition. Indeed, secretion levels of PGE2 and TGF-β by Sa-3 but not by HSC-4 were increased following erlotinib treatment (Figure 5). On the other hand, IL-4 and IL-10 levels were below levels of detection regardless of erlotinib treatment (data not shown).

### Change in PGE2 and TGF-β production from tumors by EGFR inhibitor

So far only few reports have reported the relationship between EGFR targeted therapy and TGF-β expression [12], the direct relationship between EGFR targeted therapy, TGF-β expression and antitumor T cells remains unproved. Contrary to our results, EGFR TKI was reported to decrease PGE2 production from lung cancer cells [19]. To evaluate whether increased TGF-β and PGE2 production by EGFR inhibitor is detected in a broad range of epithelial tumors or just restricted to the SCC cell line Sa-3, we evaluated these cytokine production in several tumor cell lines pretreated with EGFR TKI. As shown in Figure 5, TGF-β production following EGFR inhibition was increased in two additional cell lines (Calu1 and 5637). However, TGF-β production was decreased or unaltered in other cell lines. PGE2 production was seen in four to eight cell lines but EGFR TKI treatment was enable to increase production only in Sa-3. These results indicate that EGFR targeted therapy can alter TGF-β production differently depending on the cancer cell line studied.

### Immune regulatory effect of erlotinib is mediated by TGF-β and PGE2

**Figure 6:**
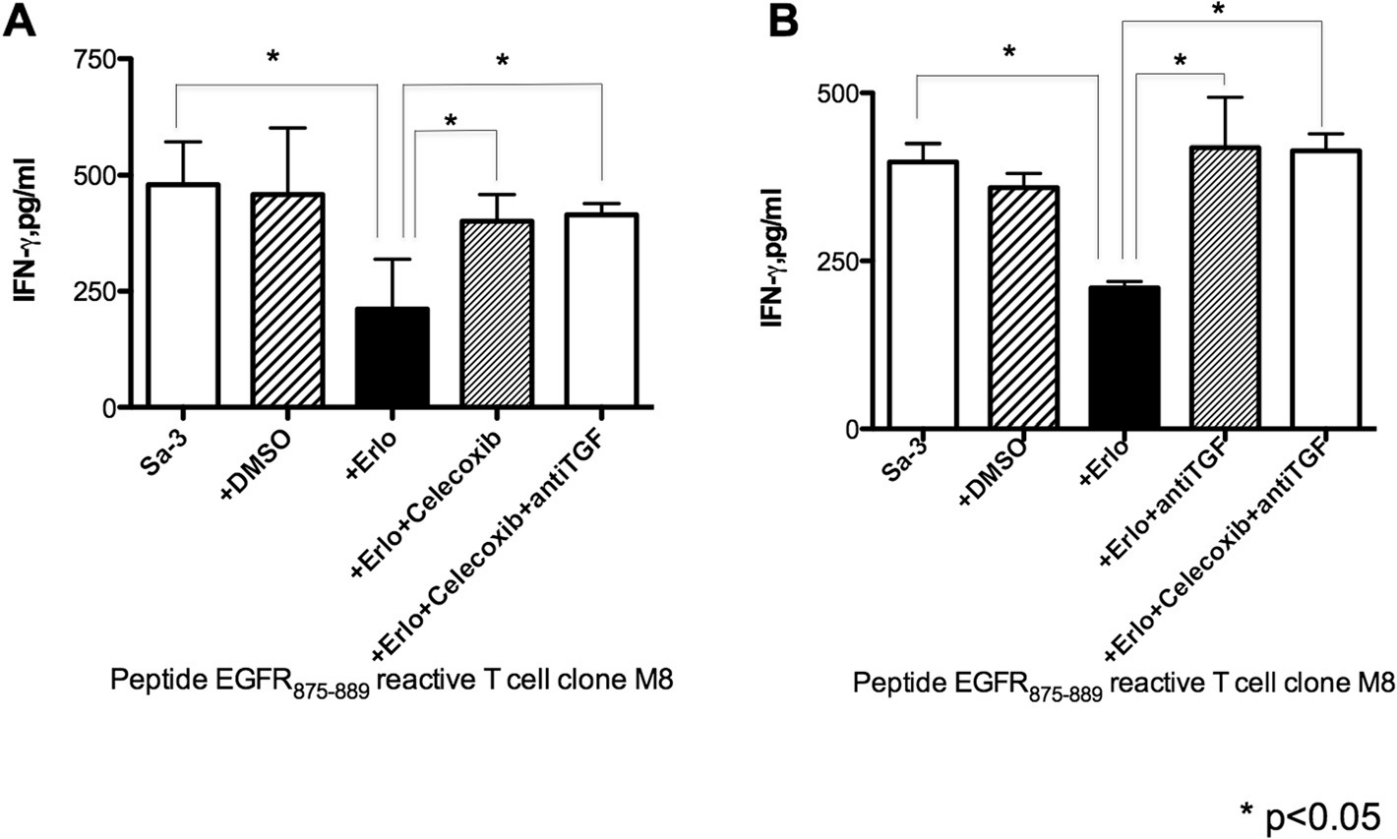
**Inhibition of COX-2 or TGF-β recovered immune-suppression by HNSCC cell line Sa-3 pretreated with erlotinib.** EGFR_875–889_-reactive CD4^+^ T cell clone M8 was tested for its capacity to recognize erlotinib (1 μM)-pretreated Sa-3 with or without **(A)** celecoxib (10 μM) and **(B)** anti-TGF-β antibody (10 μg) by quantification of IFN-γ production. Columns without bars had SD of <10% of mean values. Results are representative of three separate experiments. *p < 0.05.

**Figure 7:**
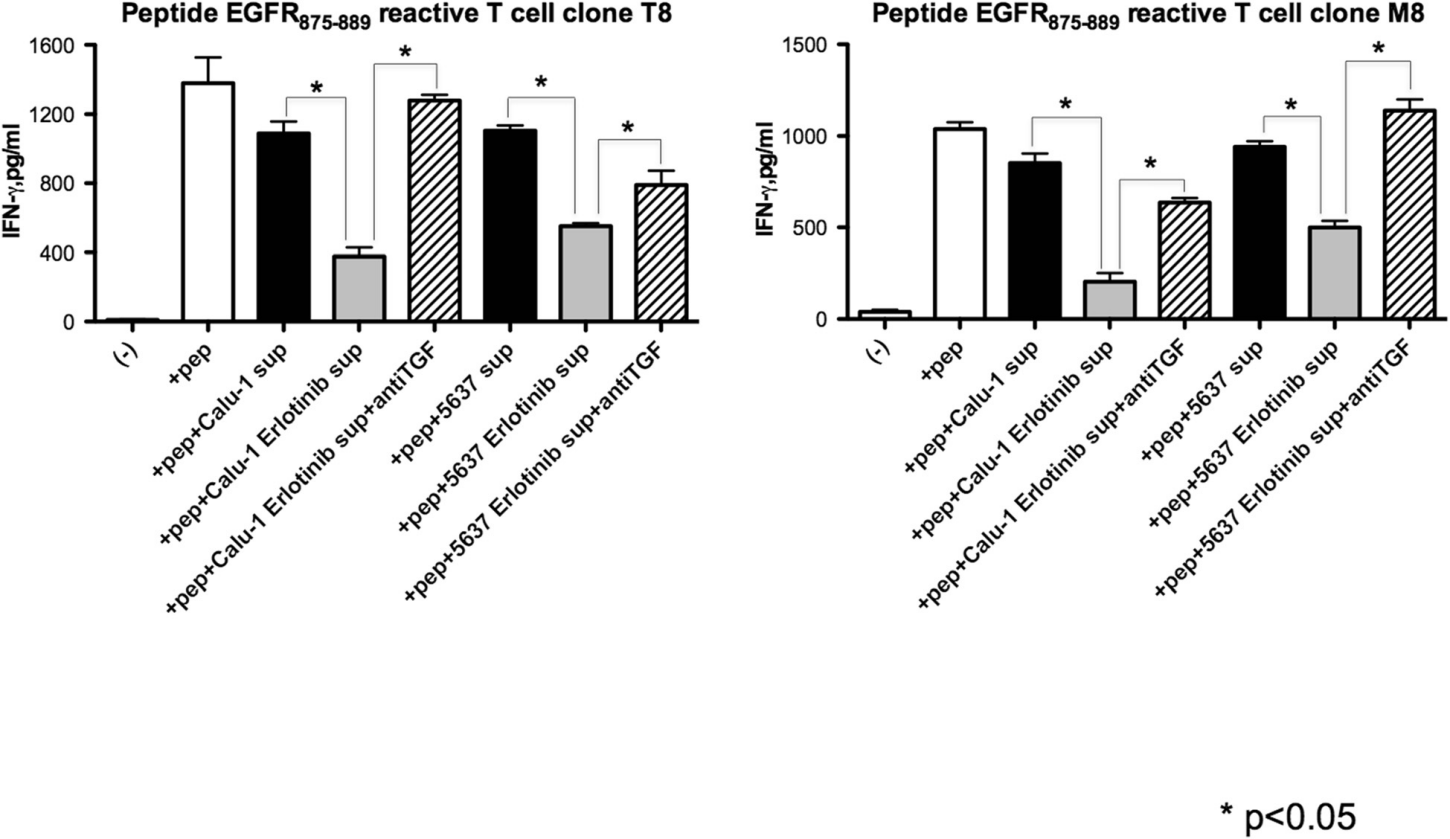
**Supernatant of tumor pretreated with EGFR inhibitor modulated T cell responses toward peptide stimulation.** EGFR_875–889_-reactive CD4^+^ T cell clone T8 and M8 were tested for their capacity to recognize 3 μg/ml peptide pulsed-autologous PBMC with or without supernatant of Calu-1 or 5637 tumor cells culture and anti-TGF-β antibody (10 μg) by measuring of IFN-γ production. Columns without bars had SD of <10% of mean values. Results are representative of three separate experiments. *p < 0.05.

The data presented above suggested that secretion of PGE2 and TGF-β by the Sa-3 tumor cell after treatment with erlotinib might be the cause of the decreased CD4^+^ T cell responses. To confirm this possibility, HTL responses against Sa-3 cells treated with erlotinib were measured following the addition of COX-2 inhibitor or neutralizing anti-TGF-β antibody. The results showed in Figure 6A show that the COX-2 inhibitor effectively restored CD4^+^ T cell function against Sa-3 pretreated with erlotinib. Similarly, anti-TGF-β antibody was sufficient to overcome the immune suppressive effects of Sa-3 treated with erlotinib (Figure 6B). Because HTLs used in this study could not directly recognize Calu1 and 5637, HTL responses to autologous PBMCs with EGFR peptide and supernatant of Calu-1 or 5637 pretreated with erlotinib were examined to strengthen the observation that EGFR inhibition mediated immune suppression through TGF-β. As shown in Figure 7, supernatant of Calu-1 and 5637 treated with erlotinib significantly reduced the HTL response and this reduction was recovered by adding anti TGF-β antibody to culture. These results demonstrate that TGF-β is at least partly responsible for EGFR inhibitor-mediated tumor immune evasion, and that targeting the TGF-β pathway and/or COX-2 pathway might be powerful strategies for restoration of immunosuppression caused by blockade of EGFR.

## Discussion

We recently reported the capacity of EGFR inhibitors to augment HLA-DR surface expression on tumor cells [8]. After HLA-DR up-regulation, most EGFR inhibitor-treated HNSCC cell lines became more susceptible to antitumor responses mediated by CD4^+^ T cells. However, in the present study, we report that despite HLA-DR augmentation on the tumor cells, in some instances EGFR inhibition suppressed antitumor T cell responses by inducing the production of TGF-β and PGE2. Suppression of anti-tumor immunity was reversed by the addition of anti-TGF-β antibody or COX-2 inhibitor, supporting the rationale for inhibition of TGF-β and COX-2 pathways to overcome potential immunosuppressive effects due to EGFR inhibition. Since EGFR inhibitor plays a detrimental role in tumor proliferation, it would be assumed that the decreased number of tumor cell could affect the immune reaction because of the lesser antigen. Pretreatment with erlotinib definitely decreased tumor cell survival in our study (data not shown), however, we did not conclude this antigen reduction is the main reason of attenuated T cell responses against tumor cells. Firstly, we washed erlotinib-pretreated tumor cells before cocultured with T cells to remove residual erlotinib. Second, we found that anti TGF-β antibody clearly recovered the function of T cells. Lastly, supernatant of tumors treated with erlotinib attenuated the T cell responses suggesting that humoral factors from tumor cells affect the T cell responses in our assay. Thus, TGF-β might at least play a harmful role in antitumor T cell responses against tumor treated with EGFR inhibitor.

In this study, we found the high variability between cell lines of expressing TGF-β and PGE2 after EGFR inhibition. Because substantial heterogeneity within tumors has been elucidated [20], it is difficult to determine a single factor that induces diversity of tumors. For example, tumor cell uses an alternative signaling such as HER-3 when EGFR signaling is inhibited [21], suggesting that tumor cells can transform their function to adapt to the surrounding microenvironment. Thus, it is speculated that TGF-β or PGE2 producing tumor cells are established from tumors that survive under immune surveillance and further studies elucidating the biomarker to distinguish the TGF-β or PGE2 producing and non-producing tumors with EGFR inhibition may help us to better treat the patients with anti TGF-β antibody or COX inhibitor.

Experimental evidences that the tumor microenvironment plays a significant role in resistance of EGFR inhibitor have been reported [12,22-25]. EGFR inhibition induces tumor cells to the mesenchymal phenotype, which cell type show resistant to EGFR inhibitor, via cytokines such as IL-6 and TGF-β [22]. Both exogenous IL-6 and TGF-β induced EGFR inhibitor resistance [12,23,24] and endogenous TGF-β was produced from EGFR inhibitor resistant tumor cells [22]. Strikingly, TGF-β receptor inhibitor abrogated motility of erlotinib-resistant tumor cells suggesting that cytokines might be promising target to overcome EGFR inhibitor resistance [25]. The TGF-β pathway is known as an important immune suppressor pathway affecting tumor microenvironment. TGF-β can regulate both the innate and acquired immune systems by inducing regulatory CD4+/FoxP3+ T cells, which represent on of the main barriers to antigen-specific antitumor immunity, and by skewing NK T cells or macrophages to regulatory phenotypes [3,4,26]. TGF-β functions not only as an immune regulator, but also as an oncogenesis promoter by inducing epithelial-to-mesenchymal transition through both Smad-dependent and independent pathways [27] or transitioning constitutive cells to a tumor-associated phenotype that facilitates tumor progression [28]. The present study revealed that TGF-β production or reduction by EGFR inhibition is depended on individual tumors. Further studies will be needed to determine whether TGF-β can function as a biomarker to assess the effectiveness of EGFR targeted therapy with and without concurrent immunotherapy.

Prostaglandins and leukotrienes are produced by the COX pathway and function as potent immune regulators. Both the tumor and surrounding stroma are capable of producing PGE2 [29], which can increase regulatory T cell activity [18]. Furthermore, PGE2 induces myeloid-derived suppressor cells, which inhibit effector T cells [30]. Accumulating evidence suggests that the application of COX inhibitors may be useful for cancer treatment both in colorectal cancer and in HNSCC [31]. In this study, we showed that EGFR inhibition augmented PGE2 production by Sa-3 tumor cell, and that COX-2 inhibitor could restore the suppression of antigen-specific CD4^+^ T cell responses. Thus, the COX-2/PGE2 pathway is partially responsible for immunosuppressive effects of tumor cells through EGFR blockade. While reduction of PGE2 by erlotinib has been reported [19], we could not detect PGE2 reduction by EGFR inhibition suggesting that the fluctuation of PGE2 production by EGFR blockade is affected by cancer heterogeneity. Recently, COX-2 inhibitor with erlotinib has been reported to inhibit the proliferation of head and neck squamous cell carcinoma in patients [32]. Although we could show the increase of PGE2 production by EGFR inhibition only in tumor cell Sa-3, we believe our result may partly elucidate the mechanism of positive effects of COX-2 inhibitor with erlotinib.

## Conclusions

We have identified TGF-β and PGE2 as immune suppressors that can disrupt EGFR inhibition-mediated immune activation. These results may facilitate strategy for targeting TGF-β and COX-2 with EGFR inhibition to overcome tumor immune evasion and reveal a novel aspect of signal targeted therapy in altering immune regulated cytokines.

## Abbreviations

APC: Antigen-presenting cell
CTL: Cytotoxic T cell
EGFR: Epidermal growth factor receptor
E:T: Effector to target
HNSCC: Head and neck squamous cell carcinoma
HPLC: High-performance liquid chromatography
HTL: Helper T cell
mAb: Monoclonal antibody
PBMC: Peripheral blood mononuclear cell
PGE2: Prostaglandin E2
TKI: Tyrosine kinase inhibitor
